# Climate Change Impacts on Mental Health Will Lead to Increased Digitization of Mental Health Care

**DOI:** 10.1007/s11920-022-01377-6

**Published:** 2022-10-10

**Authors:** Peter Yellowlees

**Affiliations:** Department of Psychiatry and Behavioral Sciences, University of California Davis, Stockton Blvd, Sacramento, CA USA

**Keywords:** Climate change, Carbon emissions, Telepsychiatry, Hybrid care, Asynchronous care, Eco-anxiety

## Abstract

**Purpose of Review:**

The evidence for the impact of climate change on the mental health of individuals and communities is reviewed, and the literature on the importance of digital systems in reducing carbon emissions is addressed.

**Recent Findings:**

Most of the climate change impacts on mental health are disaster related, although recent literature on “eco-anxiety,” often described as anxiety about the long-term effects of climate change, is emerging. There is strong evidence that the use of telepsychiatry and digital approaches to mental health care can reduce carbon emissions by reducing travel for patients and providers as well as provide effective distance care in disasters. Hybrid care, asynchronous consultations, and care at home are all innovations that will further reduce carbon emissions.

**Summary:**

The COVID-19 pandemic has rapidly accelerated the digitization of psychiatry, and climate change will continue to drive these changes in the future. Much more research on these overlapping issues is required.

*“The climate crisis is a public health and equity crisis that, absent concerted action, will continue to pose significant threats to human health.”* [1]

## Introduction

### The Impact of Climate Change on Mental Health

There have been numerous recent reviews of the severe current and future medical, mental health, and social impacts of climate change on individuals and populations around the world. Many of these reports are available at the National Academies of Medicine Climate Collaborative [2••].

Equally informative is a very comprehensive 2018 report collated by experts from eight different Federal agencies and numerous invited experts [3]. This report details the evolving health risks in seven major areas as follows:

1. temperature-related death and illness,
2. air quality deterioration,
3. impacts of extreme events on human health,
4. vector-borne diseases,
5. climate impacts on water-related Illness,
6. food safety, nutrition, and distribution, and
7. mental health and well-being.

The findings on mental health in relation to both disaster impacts and general mental health impacts were important and fascinating, with detailed descriptions of the following four key findings, all of which have substantial implications for the provision of future mental health services and how technology-related solutions used in healthcare can mitigate climate effects. Interestingly, the first two findings treated climate change as a disaster, which is where the majority of the literature has focused, while the third saw it as a cause of potential fear and future anxiety, and the fourth was a very specific comment on how heat might cause specific problems for those taking medications for mental illness.

#### Exposure to Disasters Results in Mental Health Consequences

Depending on the type of disaster, these mental health consequences include post-traumatic stress disorder (PTSD), depression, and general anxiety, which often occur at the same time. The majority of affected people were reported to recover over time, although a significant proportion of exposed individuals develop chronic psychological dysfunction.

#### Specific Groups of People are at Higher Risk

These high-risk groups include children, the elderly, women (especially pregnant and post-partum women), people with pre-existing mental illness, the economically disadvantaged, the homeless, and first responders.

#### Climate Change Threats Result in Mental Health Consequences and Social Impacts

The threat of climate change, the perceived direct experience of climate change, and changes to one’s local environment, as well as media and popular culture representations of climate change, influence stress responses and mental health and well-being. This is the first detailed commentary on what is now being called “eco-anxiety,” as discussed later.

#### Extreme Heat Increases Risks for People with Mental Illness

Increases in extreme heat will increase the risk of disease and death for people with mental illness, including elderly populations and those taking prescription medications that impair the body’s ability to regulate temperature.

The authors of the report [3] developed a helpful conceptual diagram that illustrated the key climate drivers (heat, floods, and extreme events) and pathways (levels of exposure, community displacement, and rates of death and injury) by which humans are exposed to health threats. They showed how these drivers and pathways lead to a wide range of mental health (PTSD, depression, and substance use) and well-being (resilience and post-traumatic growth) outcomes but also noted that this was all within the context of other factors that positively or negatively influence health outcomes such as resilience, access to services, and prior community engagement.

In 2016 [4], Every-Palmer and her colleagues were one of the first academic groups to exclusively focus on the climate change impacts on mental health. They concluded that extreme weather events, such as floods, droughts, heat waves, wildfires, and storms, increase the prevalence of post-traumatic stress disorder, depression, anxiety, and substance use disorders. They also described more significant indirect effects, however, that arise primarily from damage to land, infrastructure, and community functioning, leading to migration, armed conflict, and other violence. They and others [5] also noted that these effects are unevenly distributed and disproportionately affect disadvantaged people, putting those with chronic mental illness at greater risk.

**Fig. 1:**
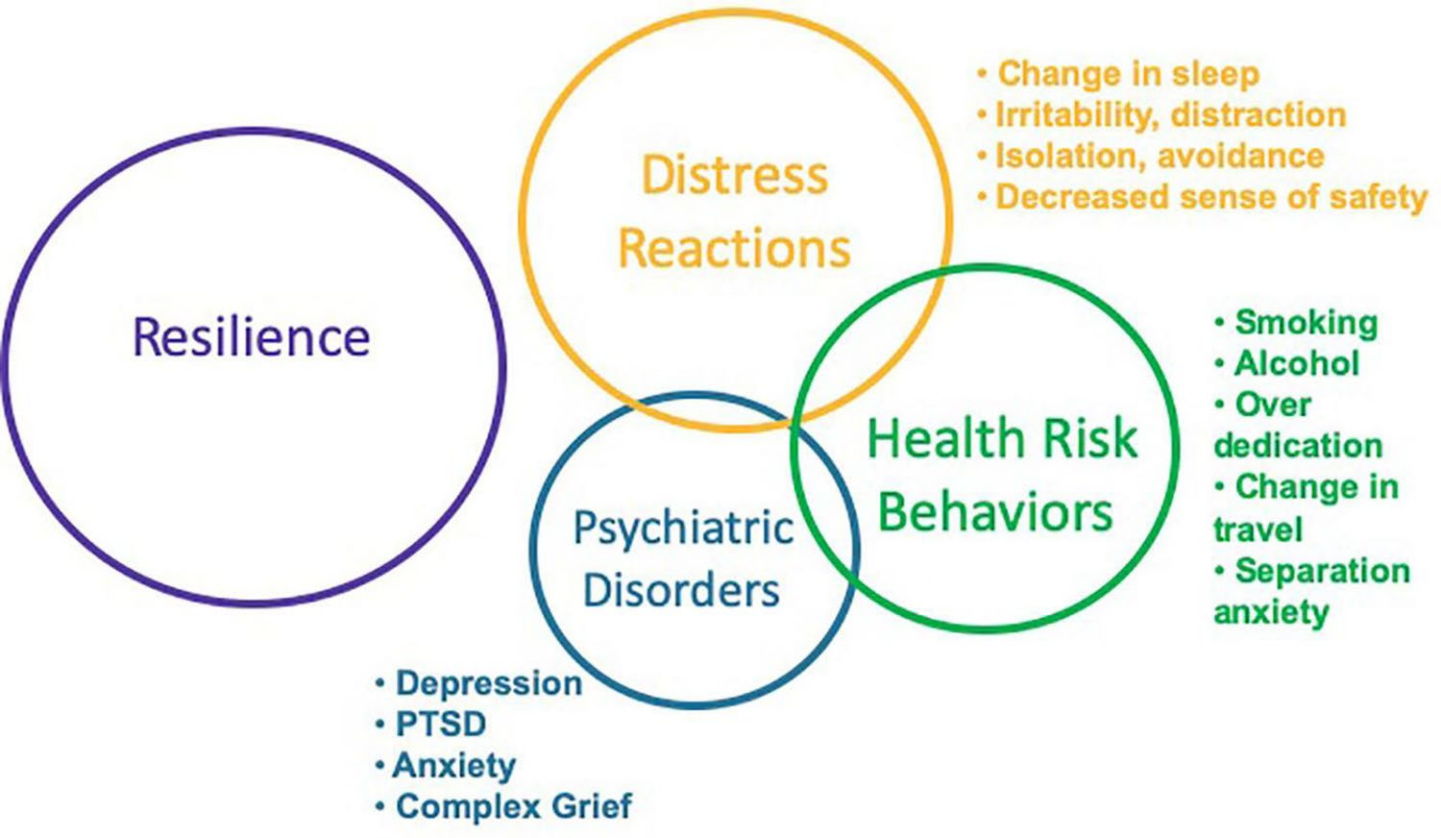
Psychological and behavioral responses to disasters [7•]

More recent publications [6, 7•] have focused on the disaster model by identifying a wider range of potential individual impacts of climate change when it is viewed as an ecological disaster, as shown in Fig. 1 (reprinted with permission from [7•]).

This disaster-focused model [7•] separates out the differing impacts of distress reactions (irritability and sleep changes) compared to health risk behaviors (smoking and drinking) and the development of psychiatric disorders (depression, PTSD, and anxiety). These three very different behavioral responses can be seen to overlap and potentially drive each other in some people. At the same time, it is clear that most people are resilient following a disaster and do not develop psychological symptoms or behavioral responses.

The American Psychiatric Association, in a 2017 position statement [8], reached similar conclusions as to which groups were most adversely affected by or vulnerable to climate change. The latter included children, the elderly, the chronically ill, people with cognitive or mobility impairments, pregnant and postpartum women, and people with mental illness, as well as people of lower socioeconomic status, migrants, refugees, and the homeless. This position statement also noted that people with mental health conditions are more likely to be affected by extreme weather events because they are more likely to be dependent upon service, infrastructure, and medication supply chains that are often disrupted after disasters, as are first responders who may be both a responder and victim required to provide care for the public while managing the adverse impacts of a disaster for their own family.

In a recent comprehensive scoping review of climate change and mental health, Charlson et al. [9••] noted how the World Health Organization [10], over a decade earlier, had proposed five global research priorities for protecting human health from climate change:

1. assessing the risks,
2. identifying the most effective interventions,
3. guiding health-promoting mitigation and adaptation decisions in other sectors,
4. improving decision-support, and
5. estimating the costs of protecting health from climate change.

This paper [9••] reviewed 120 studies published between 2001 and 2020 and concluded that climate change-related events were shown to be associated with psychological distress, worsened mental health (particularly among people with pre-existing mental health conditions), increased psychiatric hospitalizations, higher mortality among people with mental illness, and heightened suicide rates. They also noted that while climate change and mental health represent a rapidly growing area of research, such research efforts need to accelerate and broaden in scope to respond with evidence-based mitigation and adaptation strategies.

To summarize, not surprisingly, most of the literature on climate change and mental health have focused on the impact of disasters. In recent years, however, the scope of the impact literature has expanded with the introduction of terms such as “eco-anxiety,” “ecological grief,” “climate change distress,” “eco-trauma,” and “eco-angst,” to name a few. Eco-anxiety and most of the other terms refer to persistent worries about the future of Earth and the life it shelters but also acknowledge that this concern often involves symptoms beyond those of anxiety alone. Cunsolo et al. [11•], in a review article on this topic, concluded quite positively that ecological grief and anxiety over current losses or anticipated future change are a sign of relationships with, or connection to, the natural world. They noted that emotions are often what leads people to act and described how, in their view, it is possible that feelings of ecological anxiety and grief, although uncomfortable, are in fact *“the crucible through which humanity must pass to harness the energy and conviction that are needed for the lifesaving changes now required”* [11•].

Let us hope that they are right and now move on to examine how enhanced uses of information technology in healthcare may be at least a partial solution to the impending impacts of climate change.

## Solutions Involving the Digitization of Mental Healthcare

Much is known about approaches to decarbonize our planetary emissions, but surprisingly, little has been written about digital or technologically focused solutions to the climate crisis, or even more specifically toward mitigating the impact on mental health among the population groups mentioned above. As such, this section will focus on four broad areas only, in the knowledge that other technologies, especially heating, cooling, energy production, and storage technologies, not mentioned in detail, will undoubtedly become important in the future. The areas where there is evidence to support the use of digital technologies to mitigate the mental health impacts of climate change specifically are.

1. education of the health workforce about climate change and how to reduce the adverse carbon impact of health systems in general;
2. technological responses to disasters – prevention and interventions;
3. clinical practice changes using information technology that will improve the general delivery of physical and mental health services, especially to vulnerable groups, which lead to carbon savings through reduced travel of patients (and providers) when using telemedicine with patients in their homes, practicing in a hybrid manner and using asynchronous approaches to care; and
4. social media communications about Climate Change Anxiety (CCA)-related disorders

### Education of the Health Workforce About Climate Change and How to Reduce the Adverse Carbon Impact of Health Systems in General

We know that bits and bytes lead to much less emitted carbon than concrete and paper, so from a climate perspective, it is essential that our healthcare systems change and become more focused on the provision of virtual services in the future. Changing healthcare systems and educating of the workforce about climate-related topics are the primary foci of the National Academy of Medicine Climate Collaborative [2••]. The Climate Collaborative notes how climate change is not only increasingly affecting people’s health but, ironically, that health systems themselves are responsible for approximately 8.5% of US carbon emissions. The Collaborative aims to reduce this footprint and thereby gain significant national health, social, and economic benefits by educating and activating all parts of the health sector to drive sustainable change.

The Climate Collaborative represents health and hospital systems, clinicians, private payers, biopharmaceutical and medical device companies, health care services, health professional education, academia, nonprofits, and the federal government. It aims to provide a neutral platform for its participants to align around collective goals and actions for decarbonization based on evidence, shared solutions, and a commitment to improve health equity. The Climate Collaborative’s work focuses on the health care supply chain and infrastructure; health care delivery; health professional education and communication; and policy, financing, and metrics.

With this broad set of aims, it is evident that a great deal of the Collaborative’s work will involve improving the efficiency of healthcare systems at all levels and encouraging the use of multiple information technologies to meet this need. Ultimately, healthcare systems themselves have to become less focused on bricks and mortar and more involved in the provision of virtual electronic services and infrastructure. The same applies to both research and education, areas like clinical services that, during the Covid pandemic, have suddenly morphed into a greater dependence on virtual, or hybrid, approaches, and which, with the importance of climate change, need to continue in this way long term.

### Technological Responses to Disasters – Prevention and Interventions

Technology will, in the future, be commonly used to deliver care to those involved in disasters. This has been seen with the dramatic move of mental health care away from clinics to the home and to mobile devices, as seen during the COVID-19 pandemic, as discussed below. The American Telemedicine Association [12••] has developed a number of position statements and white papers over the past decade on how best to use video, audio, and other technologies to provide direct mental health care during disasters, as well as other forms of emergency medical care. The American Psychiatric Association [13] has also developed numerous guides and videos as part of its telepsychiatry toolkit for practitioners which have been widely used during the pandemic, and jointly with the American Telemedicine Association [12••] has published widely used practice guidelines.

Much has been written about the use of technology in both the prevention and the provision of care for disaster victims and first responders. Morganstein and Ursano [7•] have provided the most complete recent review of the wide range of responses to disasters including those delivered as necessary by technology. They have described the role of the media in communication to potential victims before, during, and after a disaster, and in particular how social media can be used to warn, inform, and educate. The potential downside of social media, of course, is the provision of misinformation, as has been seen occurring commonly in the Russia-Ukraine conflict, where both sides in the war have been not just fighting a physical war, but also an information war.

Just as there are many social media sites of relevance to disasters, so there are hundreds of apps focused on disaster recovery. Detailing these is beyond the scope of this article, but Morganstein and Ursano [7•] have described seven apps, mainly developed by Federal Agencies, that appear particularly useful. Between them, this small collection of apps provides access to weather services, the location of local services and shelters, response tips, information on behavioral care, sources of available energy (gas for vehicles), communication with first responders, information about potentially harmful substances, and a range of medical and recovery tips and suggestions.

Just as apps can deliver this wide range of necessary information, so can peer-responder teams use video and telephony to contact victims and provide psychological first aid, a relatively simple evidence-based approach to care for immediate victims of disasters. This can be done either fully electronically, or in a hybrid manner, with experts at a distance working with local personnel at the disaster site. For those victims who have pre-existing psychiatric disorders, then synchronous or asynchronous video or text visits can be used.

### Clinical Practice Changes Using Information Technology that will Improve the General Delivery of Physical and Mental Health Services to Vulnerable Groups and Save Carbon

Telepsychiatry has been an evidence-based practice for many years with over two decades of research and clinical guideline development supporting its use and effectiveness when compared with in-person treatments across diagnoses, settings, and populations [14, 15, 16••]. In the past decade, new models of hybrid psychiatric care have been created by blending videoconferencing across various components of health systems, including in-person care, with other technologies such as EHRs and mobile health apps using multiple passive data collecting tools on smartphones. This has led to new virtual models of integrated care and the development of asynchronous psychiatry consultations using both electronic health records and recorded patient videos. Increasingly, in the USA, from 2015 onwards, psychiatrists have been engaged in “hybrid-doctor-patient relationships,” which, post-COVID-19, are increasingly common and accepted for use for routine mental health provision as well as in disasters to assist both victims and first responders. All of these changes, with the move to treat people at home and in the community, will lead to important reductions in carbon emissions.

Three recent systematic reviews of studies of healthcare impacts on greenhouse emissions have described substantial savings as a consequence of the use of telepsychiatry and other digital technologies, leading to less travel for healthcare by both patients and providers. All three reviews [17, 18, 19••] arrived at the same conclusions, namely that the reduced travel (primarily less driving and flying), associated with digital healthcare and education, can lead to significant carbon footprint savings, with Ravindrane and Patel [17], noting that all 14 studies they reviewed show significant environmental benefits.

Donald and Irukulla [19••] identified 31 studies totaling over 57,000 patients. They reported carbon savings ranging from 0.69 kg carbon dioxide equivalent to 893 kg carbon dioxide equivalent per encounter. Distances saved also ranged from 6.1 to 3386 km. Further analysis of 18 of the included studies was conducted for cost savings that ranged from €1.73 in fuel costs to over US $900 in travel-related expenses. Similarly, 15 included studies were analyzed for time savings, which ranged from 38 min to 24 h.

In a separate major recent review of over 50,000 Kaiser Permanente outpatient visits, Wicklund [20] reported that connected health platforms dramatically reduced greenhouse gas emissions. In summary, it seems clear that any change to medical or mental health practice that leads to less travel or the use of less clinical buildings because of providers working at home (building construction leads to high carbon emissions) will lead to significant climate-related benefits. Fortunately, these are just the type of changes that are happening now in the practice of psychiatry and behavioral health.

There are three specific innovations in mental health practice that seem likely to be permanent, all of which are likely to reduce the amount of traveling by both patients and providers and thereby contribute to further reductions in atmospheric carbon.

#### Innovation 1 – Hybrid Psychiatric Care

The use of a “hybrid” model of care combining in-person consultations and telepsychiatry has been described in detail by Yellowlees et al. [21•] and Yellowlees and Shore [16••] who defined a hybrid psychiatrist as a clinician who *“ interacts with patients both in-person and online so that their doctor-patient relationship crosses both environments. The addition of interactions *via* videoconferencing, e-mail, text messaging and telephony leads to improved access and interactions at times and places not possible with care restricted to the in-person venue.”*

The American Psychiatric Association acknowledged how prevalent this style of practice has become among American psychiatrists during COVID-19 and published a guidance document for members about opening or re-opening their practice during COVID-19 [13] with the following advice: *“the safest way to continue providing treatment is through telehealth when feasible, particularly if this has been a viable option to date. Many patients may want to continue working in a hybrid way, with a mix of in-person and telehealth visits.”*

It is likely in the post-COVID-19 world that many, if not most, mental health professionals will continue to practice in this hybrid manner, seeing patients both online and in-person, depending on mutual convenience and preference. This may well lead to the development of a number of novel future psychotherapies and mental health treatments that will take the best aspects of both in-person and online models of care and therapy and combine them. In some places, this is already happening, with Fortney et al. [22] describing the use of online video sessions with veterans who have PTSD as an engagement strategy to eventually lead to them attending in-person group sessions.

#### Innovation 2 – Care Delivered from and to the Home

The introduction of smartphones and mobile devices has suddenly made it possible to easily connect with patients directly in their homes, their workplaces, social environments, and their vehicles, rather than having to see them in primary care clinics, as has been the case with traditional telepsychiatry. This saves large amounts of travel and means less need for outpatient clinic space as providers increasingly work from home, a move that is also known to improve their overall well-being.

The home offers many more opportunities to get to know patients and to further deepen understanding and the level of the doctor-patient relationship [16••]. There are many advantages of seeing patients on video in their homes or community settings besides the environmental advantage. These include the following.

- The doctor-patient relationship is likely more egalitarian, and the environment is less intimidating for patients.
- The interview can be more intimate, especially when trauma is involved, as the slightly increased virtual distance frequently makes it easier for patients to confide in their physician.
- The consultation is more convenient and safe for both parties, who both save both time and cost.
- More information can be found in the home setting which is really an extension of the mental state exam – Homes are a reflection of who we are.

Of course, the carbon advantages of seeing patients in their homes are magnified if the provider is also working from his or her home, as is now increasingly happening.

#### Innovation 3 – Asynchronous Telepsychiatry

Yellowlees et al. [23••] have recently reported on a two-year randomized clinical trial of synchronous telepsychiatry compared with asynchronous telepsychiatry in primary care patients and have found that both modalities led to similarly improved clinical outcomes, and with both approaches having positive environmental benefits. The authors noted that asynchronous telepsychiatry is a more data-rich form of the traditional medical or psychiatric “curbside consultation,” which makes use of a completely virtual care model with the potential to scale and enable psychiatrists to be involved in the treatment of more patients over a given time period using multiple technical platforms involving text, video, and audio. As such, it is of great potential interest in countries around the world, where psychiatrists are in short supply and where current carbon emissions are high, such as India and China. In recent years, asynchronous technologies have become more widespread in many healthcare settings, and such asynchronous tools have been expanding into mental healthcare, where they may be at least a partial solution to address the psychiatric workforce shortage and reduce access barriers for patients [16••]. Other positive patient outcomes have been described [24] with e-coaching for depression, mobile-based asynchronous text messaging therapy with licensed therapists, and the use of an integrated asynchronous virtual care platform.

### Social Media Communications About Climate Change Anxiety-related Disorders

CCA disorders are commonly mentioned in the social and news media, but are they really psychiatric disorders, or more of a now commonly held existential worry? And are they driven on social media by accurate information, or by misinformation, and in a way that may lead to social disruption? Little is really understood at a scientific level about these questions; few studies (almost all qualitative) have been published, and there is a dearth of useful knowledge currently available beyond editorial opinions on this topic.

Two early useful review studies do exist that have been published recently.

Schwartz et al. [25] have described and reviewed the growing body of research that has documented the phenomenon of CCA, defined broadly as negative cognitive, emotional, and behavioral responses associated with concerns about climate change. They noted that there are few empirical studies on CCA and little evidence regarding whether CCA is associated with psychiatric symptoms and specific disorders and whether engaging in individual and collective action to address climate change could buffer such relationships. They found that engaging in collective action, but not individual action, significantly attenuated the association between CCA, cognitive emotional impairment, and symptoms of depression and concluded that it appears to be important to create opportunities for collective action to build a sense of individual and community urgency in addressing climate change.

Soutar and Wand [26] have noted that the concept of CCA is poorly understood in their systematic review of the qualitative literature regarding the scope of anxiety responses to climate change, with only fifteen studies with little geographical diversity meeting the inclusion criteria. The scope of anxiety they defined included worry about threats to livelihood, worry for future generations, worry about apocalyptic futures, anxiety at the lack of response to climate change, and competing worries. Themes pertaining to responses to climate change anxiety included symptoms of anxiety, feeling helpless and disempowered, and ways of managing climate change anxiety. They concluded that there is a need for high-quality psychiatric research exploring its clinical significance and potential interventions.

## Conclusions

The mental health professions can have an impact on climate change by, among other activities, further digitizing our work. Climate change is undoubtedly the most significant current threat to global health, including mental health, impacting it at many individual, social, and community levels.

While there are many mitigation approaches being tried around the world, global warming continues, and the healthcare system itself in the USA is responsible for about 8.5 percent of all US carbon emissions. The task for healthcare professionals is to not only be educated advocates about the need for climate impact mitigation strategies generally but also to carry out more research and implement changed work practices to reduce the amount of carbon produced as we work. In the mental health area, in particular, there are a lot of changes that can occur that will reduce carbon emissions. It is evident that the practice of telepsychiatry, both synchronous and asynchronous, is one of these which will lead to less travel for both patients and providers and have the potential to reduce the mental health impact of climate change quite significantly.

There is a need for concerted action to increase the digitization of mental health care.
